# Understanding fragile X syndrome from a mother's perspective: Challenges and resilience

**DOI:** 10.3402/qhw.v11.29512

**Published:** 2016-04-20

**Authors:** Chantel Lynette Weber

**Affiliations:** Department of Psychology of Education, University of South Africa, Pretoria, South Africa

**Keywords:** Carriers, disability, impairment, parenting, well-being, South Africa

## Abstract

The purpose of this study is to communicate findings from a case study on a South African mother with three children diagnosed with full mutation fragile X syndrome (FXS). The participant is an unaffected carrier of FXS. Research has shown that mothers of children with FXS often experience high levels of parenting stress and low levels of psychological well-being. However, observations made have piqued curiosity about their positivity and determination to carry on each day raising children diagnosed with FXS. The aim is to develop a better understanding of the manner in which a mother of children with FXS make sense of her situation, to gain further insight into the specific resilience processes she acquired. A qualitative case study approach was followed, gathering data through semi-structured interviews based on open-ended questions. The findings offer new insights into a South African mother's life raising children with FXS. Even though there is very limited support and little awareness of FXS in South Africa, she still found ways to seek help, and find solutions to every day challenges. The study conclusions discourage blind stereotyping of mothers of children with FXS as vulnerable only. Future research should concentrate on promoting awareness, education, advocacy, and support for individuals with FXS in South Africa.

In South Africa, disabled individuals and their families have limited support and available resources. It is the sole responsibility of these families to care for their disabled child themselves. They are responsible for any financial costs of professional services the child might need (Greeff & Nolting, 2013). To date, literature on the experiences of parents raising children diagnosed with fragile X syndrome (FXS), as well as the processes involved to ensure resilience among these parents is limited. This study aims to add knowledge and create awareness on everyday experiences of a parent living with children diagnosed with FXS, in South Africa.

I was a live-in carer to an adolescent female diagnosed with full mutation FXS living in the United States of America in 2008. During my time as live-in carer, I was able to learn more about FXS and complete my studies concentrating on resilience and FXS. In 2014, while completing research in South Africa, it came to my attention that many professional South Africans are uninformed and unaware of this condition. I realized that there is a need to create awareness on the syndrome, specifically in South Africa.

Significant challenges are experienced by parents rearing a child with an intellectual disability (Sterling, Barnum, Skinner, Warren, & Flemming, 2012). These specific challenges may cause parents to experience high levels of emotional and personal tension, which in turn will impact on the psychological well-being of not only the affected child but the entire family unit collectively (Sarimski, 1997). However, some mothers of children diagnosed with FXS have been found to be positive and determined to carry on each day, despite the adversities they are faced with (Hauser, Kover, & Abbeduto, 2014; Poehlmann, Clements, Abbeduto, & Farsad, 2005). This prompted the following research question: What are the experiences of a mother raising children diagnosed with FXS? More specifically, this research has two objectives:

- To understand and identify specific challenges of a mother, who is an unaffected carrier, raising children diagnosed with FXS;
- To gain further insight into the specific resilience processes she acquired.

The information gathered from this research will provide parents, educators, and health care professionals with a greater understanding of how parents experience and cope with raising children diagnosed with FXS, specifically in South Africa. Improved knowledge will enable parents to relate to others in similar situations, and educators and health care professionals to be better informed and to assist parents of children with FXS.

## Defining FXS

FXS is the most common hereditary cause of intellectual disability currently known around the world (Cornish, Turk, & Hagerman, 2008; Essop & Krause, 2013; Fernandez Carvajal & Aldridge, 2011; Kesler, Lightbody, & Reiss, 2008). FXS is a genetic disorder, so called because of a fragile site on the tip of the long arm of the X chromosome where, although not quite separated, it looks as though the end is broken off.

Individuals with no obvious signs or characteristics of FXS can pass it on in a family. Both boys and girls with FXS will have a mother with a permutation or a full mutation. These women have a 50% risk of passing on the abnormal X chromosome to each child. Sons inheriting the X chromosome carrying a full mutation will have FXS, while daughters who inherit the mutation have about a 50% risk of showing features of this syndrome. For women carrying smaller permutations, the risk of expanding to a full mutation in the next generation is a little lower depending on the number of Cytosine—Guanine—Guanine (CGG) repeats. A woman who carries a FX permutation could have inherited the mutation from her mother or her father. For men with a permutation all their daughters, but none of their sons will be carriers (Hagerman, 2000; Hagerman & Hagerman, 2002; Harris-Schmidt & Fast, 2004).

## Parenting children diagnosed with FXS

Various studies have concentrated on identifying how having and raising a child with intellectual impairment affects health symptoms in parents and contribute to extraordinary challenges for them (Bailey, Skinner, & Sparkman, 2003; Hartley, Seltzer, Head, & Abbeduto, 2012; Minnes & Steiner, 2009; Smith, Seltzer, & Greenberg, 2012). According to Smith et al. (2012), their physical health, and social and emotional well-being are affected as described below.

Mothers of children with FXS were found to have more distinct headaches, backaches, muscle soreness, fatigue, dizziness, and hot flushes than mothers of children without any impairment due to the stress experienced caring for their children affected by FXS (Smith et al., 2012). High levels of depression, anxiety, and stress were reported amongst parents of children with FXS (Gane et al., 2010; Lewis et al., 2006, McCarthy, Cuskelly, Van Kraayenoord, & Cohen, 2006; Wheeler et al., 2010). Furthermore, they experienced isolation, reduced levels of parenting confidence, and feeling overwhelmed due to their children's high levels of behaviour problems (Johnston et al., 2003; Wheeler, Hatton, Reichardt, & Bailey, 2007). Parents were found to interact less and spent less time with their children and developed high levels of pessimism about their child's future (Abbeduto et al., 2004; Lewis et al., 2006).

The time of diagnosis is found to be a traumatic experience amongst parents and many reported problems in the diagnostic process, such as series of misdiagnoses, lack of support during and after diagnosis, lack of understanding, seeking supportive healthcare providers, the waiting lists to receive services, not knowing the nature of the disability, lack of knowledge and information available, interest in learning of healthcare providers, and the fact that they were often left to their own devices (Minnes & Steiner, 2009). A study done by Baker, Seltzer, and Greenberg (2012) reported that behaviour problems of individuals with FXS had a negative impact on parents’ marriages. Furthermore, the diagnosis of FXS has been found to cause families’ excessive financial burden, and impact their employment. This in turn results into a lowered quality of life and takes a prolonged toll on family finances (Wong, Mailick, Greenberg, Hong, & Coe, 2004). Furthermore, it takes a toll on family interactions and communications (Baker et al., 2012).

## Resilience as coping mechanism

In order to be seen as resilient, it is necessary to experience risks (Luthar, Cicchietti, & Becker, 2000). Risk refers to characteristics, traits, or experiences that predicts negative outcomes (Wright & Masten, 2006) and can be present in individuals (intrapersonal), their families, as well as their environments (interpersonal) (Boyden & Mann, 2005; Mash & Wolfe, 2005). Caring for children with a mental impairment such as FXS denotes risk as discussed above.

Resilience is seen as a process that involves transactions between the individual and his or her social ecology (Sameroff, 2010; Ungar, 2011). In order to acquire resilience, the individual's environment needs to make resources available, but also the individual should navigate towards and make use of these protective resources (Ungar, 2010). According to Kumpfer (1999), to be considered to be resilient, involves the following stages:

- The individual experience challenges.
- There are certain resources available in the environment.
- The individual interact with the environment.
- The individual's internal resources, such as spiritual, intellectual, social, physical, and psychological skills, buffer against the challenges.
- The individual develop the necessary coping skills to cope with the challenges she may experience.
- The individual achieve the positive outcome.

These stages support and are in line with the understanding of resilience as an ecologically embedded bi-directional process (Lerner, 2006). In other words, resilience is seen as a continual cooperative transaction between the individual and her context, influenced by her ecology (Lerner, 2006; Ungar, 2011).

A brief review of the protective resources found in previous studies focusing on resilience in family members of children diagnosed with disabilities is provided, as they align well with the context of my current study. Protective resources refer to processes that encourage resilience and influence an individual's ability to function resiliently. According to Ungar (2004), protective resources refer to variables that decrease the impact of challenges an individual is faced with and therefore reduce the possibility of negative outcomes. When referring to protective resources, they are grouped as intrapersonal resources and interpersonal resources. It is important to remember that protective resources alone do not guarantee resilience (Rutter, 1984), but the processes rooted in these resources (Unger, 2011).

Individual protective resources typically include attractiveness, assertiveness, autonomy, internal locus of control, intelligence, empathy, outgoing temperament, optimism, self-awareness, peacefulness, positive self-esteem, self-efficacy, social competency, and sense of humour (Carter, Martinez-Pedraza, & Gray, 2009; Ekas, Lickenbrock, & Whitman, 2010; Knestrict & Kuchey, 2009; Kuhn & Carter, 2006; Kumpfer, 1999; McMurray, Connolly, Preston-Shoot, & Wigley, 2008; Masten, Cutuli, Herbers, & Reed, 2009).

Protective resources within the social ecology typically include family connectedness (Bayat, 2007; Breitkreuz, Wunderli, Savage, & McConnell, 2014; Greeff & Nolting, 2013), social support (Breitkreuz et al., 2014; Ekas et al., 2010), financial resources (Breitkreuz et al., 2014), durability of family (Greeff & Nolting, 2013), access to mentors (Masten et al., 2009), communication (Greeff & Nolting, 2013), and religious beliefs and spiritual resources (Bayat, 2007; Breitkreuz et al., 2014; Ekas et al., 2010; Greeff & Nolting, 2013; Masten & Wright, 2010).

Despite the limited nature of what we know about the resilience of parents caring for children with FXS, resilience-focused studies have encouraged more positive understandings of how parents of children with disabilities cope. Although I found some studies on resilience in generalised contexts of parents of children with disabilities, I could locate only two studies focusing on the well-being of parents of children with FXS (Hauser et al., 2014; Poehlmann et al., 2005). Furthermore, only one study could be found in relation to resilience found in an adolescent with FXS (Fourie & Theron, 2012). I therefore saw this as an opportunity to explore a South African mother's experiences raising children with FXS and exploring what contributed to her resilience and making this public.

## Method

### Study design

The research objective was to study a single subject in South Africa over time and to collect detailed information about the participant (Creswell, 2013; Mertens, 2010). Therefore, a qualitative, single case study research design was followed (Creswell, 2009), anchored in the interpretive paradigm (Merriam, 2009; Stake, 1975). Making use of an interpretive paradigm allowed me to try to understand the experiences of the mother raising children with FXS, through the meanings that she assigned to them (Merriam, 2009; Nieuwenhuis, 2007a; Stake, 1975). In other words, I interpreted what the participant told me during the data collection process, and realized that what she told me is her interpretation of reality. This perspective located my work in the post-modern realm (Nieuwenhuis, 2007a). It helped me to better understand the participant's experiences, making sense of her struggles, and determining the processes that allow her to cope with her daily challenges. Case studies such as these (Mertens, 2010) can offer rich contributions to theory and practice, which strengthened my motivation to report this case. Descriptive and in-depth data were collected in order to gain greater understanding of the participant's life and experiences (Hays & Singh, 2012).

### Instruments

The data were collected by means of interviews and in situ observations. Observations were made during informal meetings as well as during the interviews, and were recorded in a reflection journal (Merriam, 1998). The research journal helped to enhance the trustworthiness of the findings (Nieuwenhuis, 2007b). I interviewed the participant twice and made use of semi-structured interviews, based on open-ended questions. Semi-structured interviews were used as it is characterized much like a conversation that is flexible and exploratory (Merriam, 1998).

Based on an eco-systemic understanding of resilience (Schoon, 2006; Ungar, 2011), I included questions about how raising children with FXS impacted the participant's life and what resources (intra- and interpersonal) appeared to buffer these impacts. The interviews were recorded in order for it to be transcribed and analysed.

### Ethical considerations

Before the study commenced, the ethics board at the University of South Africa at which I am a post-doctoral fellow provided ethical clearance (reference number—2014MAY/52287521/MC). The participant was not harmed in any physical or emotional manner. All possible or adequate information on the goal of the investigation; the procedures that were followed during the investigation; the possible advantages, disadvantages, and dangers to which the participant might have been exposed to; as well as my credibility was disclosed to the participant. The participant was given information about what the study entails and what would be expected from the participant. This allowed her to make a voluntary decision to take part in the study. She was able to withdraw from the study at any time.

The participant was observed and interviewed in her natural setting. I explained to the participant that emotions, such as uneasiness, might be experienced as she recalled previous unpleasant experiences. The participant’s real names were not used. I debriefed the participant after the interviews and observations were made. Debriefing can be defined as sessions, after the data were collected, where the participants have the opportunity to work through experiences and their outcomes (Strydom, 2005). This occurred after the interviews were transcribed rather than after analysis (Houghton, Casey, Shaw, & Murphy, 2013). The reason for doing so was to allow the participant to acknowledge and respond to her own words before they are scientifically analysed. The participant had no concerns or reservations about the content of the interview. The transcripts therefore remained unchanged and did not influence the analysis and interpretation of the data.

### Trustworthiness

To ensure trustworthiness, peer debriefing was used, where the research process and findings were reviewed and discussed with unbiased colleagues (Shenton, 2004). The credibility of the findings was checked by means of confirming with the participant if what I understood was correct (Lincoln & Guba, 1985; Mertens, 2009).

Due to the fact that I possess specific knowledge on the research subject, it influenced the choice of design and interpretation and conclusion of results to some degree. I knew what I was looking for and was able to be more focused during the data collection process. The literature review allowed me to apply logical reasoning and interpretation of the data collected (Shenton, 2004). Direct quotations from the interviews that were conducted were included. During the interview process, regular reflexivity took place and was recorded in a research journal. I noted my developing argument, feelings, and interpretations, and reflected on my situatedness in this study. Furthermore, a thick description of the participant and her context to facilitate transferability was provided. Although this does not reduce the limitations of case study methodologies, the working hypothesis that emerges from this study guides transferability.

### Participants

#### Sampling

Purposive sampling took place, which allowed me to choose a participant from whom most could be learned. A paediatrician with a lot of knowledge about FXS in South Africa was contacted. A meeting was held between the paediatrician and myself, where I explained the needs of the study, in order for the paediatrician to identify a mother of a child or children with FXS in South Africa. The participant had to be coping resiliently with her circumstances (raising children with FXS) and be willing to participate in the study. FXS is fairly unknown in South Africa. It is therefore not as easy to identify people affected by this syndrome. However, the paediatrician identified the participant as resilient. The paediatrician and I met in order to discuss how she understood and defined resilience. She defined resilience as follows: “The ability of an individual to adapt to or confront stresses or adversity of many different natures. Being able to overcome and successfully live with either psychological or physical challenges.” I discussed with her how I understood and defined resilience and together we agreed on a definition for resilience: “Resilience refers to a process between an individual, her family, community and culture. Even though the individual have faced significant challenges raising children with FXS, she has overcome the risks and demonstrated positive adaptation.” The results of the study were therefore more precise as we identified a resilient participant, rather than just randomly choosing a participant. The most could be learned from her.

The sample size of the study consists of one unaffected carrier, a mother with three children diagnosed with FXS. The reason for including only one participant is that she was the only participant the paediatrician recommended. I saw this as an opportunity to start creating awareness on FXS. The participant was protected from harm during the identification and selection process by first consulting with the identified paediatrician with FXS experience. Once the participant agreed to take part in the study, she was contacted to set a time and place convenient for her to meet.

### Data analysis

The data that were collected in the study was analysed using deductive qualitative analysis (Miles, 2014), which means that prior reading led me to identify topics that could be illustrated by appropriate quotations. The transcribed interviews were read through to get a global impression of the context. I categorized the topics according to the themes that emerged during my literature review of writing up my doctoral thesis, which shed light on resilience as well as explained the challenges the participant experienced raising children diagnosed with FXS. An independent coder (Nieuwenhuis, 2007b) was used to justify the themes that were found during analysis of the data (Creswell, 2003). Following the constant comparative method (Merriam, 1998), I made use of observation notes to support what was found in the interviews.

## Findings

The participant is an Indian mother of three children diagnosed with full mutation FXS, living in Gauteng, South Africa. She is the carrier of the syndrome that was passed on to her and her sisters from their father. She is not intellectually affected by the carrier status. She works full-time as an admin officer at a company based in her community.

Her children are aged 23 (female), 20 (male), and 16 (female). The two females are not as severely affected as their brother, as males are often more severely affected than females (Saunders, 2000). The 23-year-old female is currently attending a beauty college where she is taught certain skills that will enable her to find a job after college. The 20-year-old male and 16-year-old female are still attending school (a special needs school). The family first found out about FXS in 1997. At this time their eldest daughter just started school (and some delays were noticed by teachers). However, the reason for concern was the development of their son, who was four at this time and had not met certain important developmental milestones such as talking. A diagnosis was then made amongst all three children by a paediatrician.

She has limited access to support. Although her immediate and extended family offered psychosocial support, the community was not as accepting of people with disabilities. In order to receive grants from the government, she had to educate numerous personnel about what FXS is and what their needs are. Often she was sent away as the government officers did not know about FXS and therefore did not see it as a relevant disability. During the time of the interviews, she did not receive any grants for her children from the government.

Risks refers to characteristics, traits, or experiences that predicts negative outcomes (Wright & Masten, 2006) and can be present in individuals (intrapersonal), their families, as well as their environments (interpersonal) (Boyden & Mann, 2005; Mash & Wolfe, 2005).

As stated before, risk is a prerequisite to resilience, and can be defined as intrapersonal (present in the individual) as well as interpersonal (present in families and their environments) characteristics, traits, or experiences that most likely will result in negative outcomes (Boyden & Mann, 2005; Mash & Wolfe, 2005). As the primary focus of this study is to understand the experiences of a mother raising children with FXS, findings are used to highlight the challenges, as well as the resilience processes the participant acquired to cope with her challenges. The findings are grouped into intrapersonal and interpersonal challenges and protective resources.

### Challenges

Throughout the interview, it was found that the participant experienced many challenges. Some were related to her emotional well-being, her family, and her community. The challenges that emerged in the interviews will now be discussed.

### Intrapersonal challenges

The first intrapersonal challenge that was identified during the interviews was feelings of guilt. In the following extract the participant reported blaming herself for passing along the FXS gene:

It was hard. It was like, I used to like, uh, blame myself. A lot of times, blame myself.

When asked if finding out about FXS before starting a family would have influenced her decision making about having children her response was:

There were times I said I would not have had children.

The participant furthermore reported feeling helpless and alone, which can result in or contribute to depression and stress:

I cried and I said, is it so difficult for people to actually understand that there is a problem and I need this help.

The participant reported feeling worried about the future of her children:

Why it is so hard for me to let go… My son is finishing school now. Next year. So now, we have to now, make a plan… I mean, my kids are big. I have to look at now, what is he going to do after school.

She was often the one who had to explain to others what FXS is and what it entails:

I had to tell them, listen, this is fragile X, this is the chromosome story… So I sat with him like Dr J. did and I said to him this is what I'm talking about, this is what she's got.

This was a frustrating experience for her. Furthermore, the diagnostic process was a difficult and time-consuming process:

It is not easy for someone to go through that… I always say, umm, what I went through; I don't think everybody can handle that. It is very, very difficult. It is very, very challenging … It took a while.

The participant reported that it took a long time to receive the correct diagnosis. Furthermore, she reported that it was and still is frustrating that people are still unaware of FXS and that it is her responsibility alone to explain to others what the syndrome is all about.

### Interpersonal challenges

#### Challenges within the family

Challenges that emerged within the family were related to a feeling of isolation, relationships being affected negatively, and the financial impact that raising children with FXS has on a family. During the interviews the participant reported that she felt that her family was different than other families, that her children did not have many friends, and that socializing with peers is a struggle for her children and indirectly affects her:

They (my children) live a bit of an isolated life, because I always kept them for myself. I did what I needed to do to help them by to the best I can. But as far as going out and being with people and socialising, I didn't do enough of that. And that, it think, that, it was like not good … she did not learn how to socialise.

The participant therefore started isolating herself and her family over the years.

Furthermore, she reported how the diagnosis of FXS had a financial impact on the family:

But it was a lot. It was time consuming. Cause I was home with him (my son). So I couldn't work. So it was like all the time, home just to see hospitals or to see doctors and therapy. And then obviously, financially it does, because, it is not just going there. It is also to take off like, with him, a lot of time from work.

#### Challenges within the community

The challenges that emerged within the community were lack of knowledge and understanding, lack of support groups, and lack of help from the government.

The participant reported saying that it was frustrating for her that very few people knew and still know about FXS. She has taken it upon herself to explain to others:

I hope and pray he understands… We need to create that awareness… people never knew… It was difficult, because people did not understand … No, and even still, even still, they don't know … I had to tell them, listen, this is fragile X, this is the chromosome story… You need support, yes … But in our case we had a little of it.

The participant joined a support group for mothers of children with special needs, and although this should have been a positive and supportive experience and resource for her, her experience was negative. This is illustrated in the following extract from the interviews:

It is not easy for someone to go through that. You need support… even with the group we had, there was no kids there that had fragile X. It was just my kids. … The other support groups they were of different disabilities.

The fact that the South African community is still fairly unaware of FXS, and limited support is provided from the government posed as a challenge to the participant, as more support would be helpful:

I had to take her and put her in a special school and I had to get funds for it and uh, because they didn't know, they were not as supportive about it … Because what I also notice is that, with a disability, if the parents are working. The government here will not give it. Ja, like uh, you have to earn a certain bracket.

I recorded the following in my research journal:

I have just met with the mother at their family home. They live in a small house in a relatively poor neighbourhood. Both parents work full-time to provide for their children, and with little support, if any from the government I am not sure how they are able to provide additional care or professional services for their children to ensure they reach their full potential.

### Protective resources

Even though the participant faces so many adversities, she still manages to generate her own protective resources, which allows her to cope with these struggles. These protective resources will now be discussed.

### Intrapersonal protective resources

The following intrapersonal factors were identified:

#### Tenacity

The participant demonstrated tenacious behaviour, as she was able to remove herself from any negative thoughts, change her thoughts to be positive, and continue each day as best she can. The following extract demonstrates her tenacity:

I did what I needed to do to help them by to the best I can … And I thought no, just let me deal with what I have to deal with. My children, my family. And I did it that way … You remove the negativity and put positive in there. And you move on. Every day is not going to be the same, in anybody's life. Any normal person. But you just have to be strong.

#### Empathy for others

The participant demonstrated empathy for others and the desire to assist others where they needed help:

I just want to help everybody. Not just kids like mine, but everybody. I have that thing.

I noted the following in my research journal:

The first impression of the participant is that of being extremely caring and wanting to bless others. I have only spoken to her over the telephone, and today was our first time that we met. When I entered the house I was greeted by the whole family. She prepared lunch for us, although I insisted that she go through no trouble as I only wanted to meet with her shortly. She said that I travelled far and that she would not feel comfortable having me travel so far and not providing lunch. It was lovely having lunch with her and her family.

#### Self-awareness

The participant was able to identify her shortcomings in raising her children, and was able to concentrate on the positive in the midst of the negative:

I always kept them for myself. I did what I needed to do to help them by to the best I can. But as far as going out and being with people and socialising, I didn't do enough of that. And that, it think, that, it was like not good. It was like, I used to like, uh, blame myself. A lot of times, blame myself. Things that you have to deal with, personally. … You will come home and you feel all down and out.

#### Self-esteem

The participant demonstrated a sufficient amount of self-esteem:

What I went through; I don't think everybody can handle that … I did what I needed to do to help them the best I can … I look at my kids, I would say, you know what, I am blessed.

#### Internal locus of control

The participant was able to have some extent control over her situation and believes that she has an influence over her own success. This is illustrated in the following extract:

I'm trying very, very hard to look in the positive…. You remove the negativity and put positive in there. And you move on. Every day is not going to be the same, in anybody's life. Any normal person. But you just have to be strong.

The following was recorded in my research journal:

From my experience interviewing other mothers of children with FXS, they all were emotional when talking about their experiences. Although she reported that it affected her, she never showed any sign of negative emotions. It seemed that she had incredible internal locus of control.

#### Positive outlook

The most pronounced attribute that the participant demonstrated was her positivity. In the next extract it is clear to see that the participant have a definite positive outlook on life:

So you look at the positive light. But I'm trying very, very hard to look in the positive … I am blessed. Because they can walk, they can do things for themselves. All these things … And I thought no, it is not so bad. So ja, I think that helped … I think, you just have to be that, the positive, the positive.

### Interpersonal protective resources

The following interpersonal factors were identified:

#### Supportive family members

In the interviews, the participant told of how stressful it was that her eldest daughter is getting married. Apart from the fact that she has a hard time letting go of her eldest, she furthermore worries about the child's impairments and how the new family by marriage will cope and deal with her condition. In the interviews she told of how supportive her extended family members were when she told them about the proposal:

… the nice thing was you can see the family support you know, because they were so supportive. They all came, and when we mentioned it to them they were all excited. “Can we phone, can we help, can we do this?”

I was able to observe how supportive her extended family was:

During the interview, her husband returning from work interrupted us. His brother and his nephew, who offered him a ride home from work, accompanied him inside the house. Furthermore there seemed to be a close relationship between the affected children and the visiting cousin.

The participant told of an incident where an aunt was encouraging and supportive during a special event where her son gave a speech (that wasn't planned). She was worried that people would not understand that he has an intellectual impairment. Her aunt encouraged her to relax and allow her son to take part:

And then my aunt was there, she was sitting next to me. And then she told me, no just leave him. Let him do it. Even if he does it wrong. Just let it be … And my aunt was like in tears because, she was like, ah, you are so blessed.

#### Marital quality

During the interviews I asked the participant where she receives the strength to carry on each day. She replied:

From family. But more just the two of us.

From this statement, it is clear to see that the participant and her husband have a close relationship. She furthermore reported:

I think because of the love … I think, you just have to be that, the positive, the positive. Positiveness and the support. It is like, if I didn't have his support (her husband).

#### Family time

The participant reported that spending quality time together as a family was a blessing:

God has blessed me however he has. He had a reason to give me what the kids have and I am really blessed, because I had the time with them. Every bit of the time … you know spending quality family time.

She and her family enjoy spending time together and she values this time as a family.

#### Supportive community

Even though it has been reported that support from the community is very limited, the participant mentioned several supporting factors within her community. Her experience was that her and her husband's working community was very supportive when it came to taking time off to take the children to doctors’ appointments:

And the good thing is that wherever he worked he would tell you they were really supportive. Even with this job, he got. And now that I work, they really were understanding. They understood that I needed to take them to hospital and why I had to go and that kind of thing.

The participant reported that others had helped her to get information on FXS in the early years when there were very limited resources:

And then what happened was umm, he (her husband) had friends. That when he spoke, they went and like they did resource on the internet. And then I used to go to the library and then get books and then read. That also helped. That made a big, big difference.

#### Role models

The participant reported that she had a close relationship with a psychologist, who also had special physical needs. She stated that even though this individual faced many physical adversities, she was always positive and determined to carry on. The participant reported that seeing this role model cope so well, created a sense of determination within the participant to carry on each day, and made the participant concentrate on the positives in her life, such as her own health:

But I know the lady I told you that passed away, remember, she was very good, she was very good … cause she also had a disability. And looking at her made me appreciate life and made me realise that if she can carry on each day, so can I.

Furthermore, it was found that a paediatrician (who made the diagnosis) was very supportive. The paediatrician explained to the family where necessary, and gave them advice when needed:

Cause I explained to her my family situation. Then she said, okay I think it is this. Then she spoke to my aunt, she spoke to the school that my eldest daughter goes to and from there that helped her to get her resources more correct. Then when she did that, uh … then she referred us to a doctor … You know she told us what we can do and whatever.

#### Resources

The participant's experience is that there are not many resources available to individuals diagnosed specifically with FXS in South Africa. However, the participant reported that some of the medical expenses are free and that this helped tremendously:

Okay, with my youngest daughter we don't pay. With Dr J., even till now, we don't pay … The only place you don't pay is Dr J., at the hospital. And the government hospitals for them, because they have a disability.

#### Religious beliefs and spirituality

The participant demonstrated strong religious beliefs and spirituality:

but at the end of the day it is up to God. A doctor can tell you that, that is the possibility, but when god walks, he walks differently. So you look at the positive light.

She felt that as a parent, she's been chosen to raise these special needs children and that if she wasn't able to do so, God would not have given them to her to rise:

If god didn't want us to have these kids, he wouldn't have given it to us. I think he has chosen us.

She feels blessed that God trusted her with the important task to raise special needs children, where others might feel cursed:

God has blessed me however he has. He had a reason to give me what the kids have and I am really blessed, because I had the time with them … at the end of the day you have got the creativer.

It is clear to see that numerous factors within the participant's immediate, as well as environment around her were found to influence the way in which she copes with her everyday challenges.

## Discussion

The challenges identified in this study relating to a mother raising three children which are diagnosed with full mutation FXS, builds on the broader literature that details experiences of parenting children with disabilities. The participant was able to identify numerous challenges that she experienced raising children with FXS, including intrapersonal challenges as well as challenges within her social environment. The intrapersonal challenges that came forth in the interviews were feelings of guilt and blame, concerns about reproduction, feeling helpless and alone, feeling worried about the future, the burden of educating others, and challenges during the diagnostic process. The interpersonal challenges that were identified were the feeling of isolation, negative impacts on relationships, financial impacts, lack of knowledge and understanding among the community, lack of support, and lack of help from the government. However, the participant was able to overcome the adversities and cope well with her challenges.

The participant's tenacity, empathy for others, self-awareness, high levels of self-esteem, internal locus of control, and positive outlook guided her towards resources and experiences that supported her. At the same time, her protective social ecology (supportive family members, stable and supportive marriage, quality family time, supportive community, community resources, and religious beliefs and spirituality) nurtured and enabled her to cope with her daily challenges. These resources created the opportunity for her to experience acceptance, which resulted in a positive outlook on life, and having a sense of purpose and contentment. In essence, these protective processes that were identified conceptualises resilience as a supportive person–context transaction (Lerner, 2006; Ungar, 2011). It therefore demonstrates that positive adjustment depends on both the individual and the individual's social ecology to ensure health-promoting results (Ungar, 2011).

Therefore, interactive protective processes, such as interpersonal agency, social support, and religious beliefs and spirituality encouraged the participant's resilience. Her ability to navigate towards resources and resources being available buffered her, and therefore promoted her positive adjustment to the daily challenges of raising children with FXS. Furthermore, the research findings clearly indicate that the mother's ability to cope under difficult circumstances is not merely personality traits, but an ecologically embedded process that includes interpersonal as well as intrapersonal resources.

The current research only focused on a single case study, which contributes significantly to our current knowledge because it is the first case reported in South Africa and more specifically includes a mother who had three children before the diagnosis were made. The lack of professional and general knowledge of FXS that was experienced by the mother in this case clearly indicates that more research is necessary, and that the sample be expanded to more cases, including the fathers of children with FXS. Research should focus on all ethnical groups in order to optimize the support and other factors in South Africa and cross-cultural understandings of parenting children with disabilities.

This study does not only concentrate on the negative aspects of raising children with FXS but also offers a positive perspective on raising children diagnosed with FXS. Mothers of children diagnosed with FXS have historically been considered in terms of their vulnerabilities as carers for children with impairments, as well as the results of being carriers of the syndrome (Johnston et al., 2003; McCarthy et al., 2006; Ouyang, Grosse, Raspa, & Bailey, 2010; Sterling et al., 2012). Although not all mothers are like this participant and furthermore raising children with FXS is often a challenging experience, reporting this mother's ability to rise above her circumstances resiliently suggests a transformative perspective (Mertens, 2009). This study does not only concentrate on the negatives and therefore discourages seeing mothers of children diagnosed with FXS as at risk only.

## Conclusion

This study contributed to literature as it specifically concentrated on a mother of three children diagnosed with FXS. It provides insight into the challenges and the process of resilience in mothers of children with FXS. We now have a better understanding of the challenges the participant faces raising FXS children in South Africa, and the resources that have helped her to be where she is. This understanding will enable and allow parents in similar situations to relate her experiences, and educators and health care professionals to be able to assist parents of children with FXS accordingly. This study therefore discourages negative stereotyping of mothers of children with FXS and hope to encourage communities, and other families to partner with families affected by the diagnoses of FXS, in order to work in partnership with them to promote their resilience.
